# The Effect of a Vegan Diet on the Cardiovascular System

**DOI:** 10.3390/jcdd10030094

**Published:** 2023-02-22

**Authors:** Michail Koutentakis, Stanisław Surma, Sylwester Rogula, Krzysztof J. Filipiak, Aleksandra Gąsecka

**Affiliations:** 1 1st Chair and Department of Cardiology, Medical University of Warsaw, 02-097 Warsaw, Poland; 2 Faculty of Medical Sciences in Katowice, Medical University of Silesia, 40-752 Katowice, Poland; 3 Institute of Clinical Sciences, Maria-Sklodowska-Curie Medical Academy, 00-001 Warsaw, Poland

**Keywords:** vegan diet, plant-based, health benefits, nutrients, cardiovascular health, CVD, risk factors

## Abstract

The vegan diet, often known as a plant-rich diet, consists primarily of plant-based meals. This dietary approach may be beneficial to one’s health and the environment and is valuable to the immune system. Plants provide vitamins, minerals, phytochemicals, and antioxidants, components that promote cell survival and immune function, allowing its defensive mechanisms to work effectively. The term “vegan diet” comprises a range of eating patterns that prioritize nutrient-rich foods such as fruits and vegetables, legumes, whole grains, nuts, and seeds. In comparison to omnivorous diets, which are often lower in such products, the vegan diet has been favorably connected with changes in cardiovascular disease (CVD) risk markers such as reduced body mass index (BMI) values, total serum cholesterol, serum glucose, inflammation, and blood pressure. Reduced intake of low-density lipoprotein (LDL), saturated fat, processed meat, and greater consumption of fiber and phytonutrients may improve cardiovascular health. However, vegans have much smaller amounts of nutrients such as eicosapentaenoic acid (EPA) and docosahexaenoic acid (DHA), selenium, zinc, iodine, and vitamin B12, compared to non-vegans, which may lead to detrimental cardiovascular effects. This review aims to present the effect of plant-based diets (PBDs), specifically vegan diets, on the cardiovascular system.

## 1. Introduction

Plant-based diets, which include semi-vegetarian and vegetarian diets, emphasize cereals, fruits, vegetables, legumes, and nuts while limiting animal-based foods such as meat, dairy products, and eggs. In a plant-based diet, the level of animal food limitation varies greatly. There are several types of vegetarian diets, such as the vegan diet, which excludes all animal products, including eggs, milk, and milk derivatives, ovo-vegetarians who consume no animal meat or bioproducts besides eggs, and the lacto-ovo vegetarian diet, in which eggs, milk, and milk products are included, but no meat is consumed. There are also pescatarians, or people who eat plant-based diets that include dairy and fish [1,2].

Veganism seems to be the most stringent of all the plant-based diets since it excludes all animal-related substances [3]. A whole-food plant-based diet (WFPBD) is another sort of vegan diet that has demonstrated substantial advancements in cardiovascular health, diabetes, and cancer types [4]. More specifically, WFPBD features many fruits and vegetables, whole grains, legumes, and natural soy products while eliminating animal products, processed carbs, fat, and sugar.

The long-term impacts of plant-based diets on health outcomes may be difficult to disentangle from plant-based diet-associated behaviors (e.g., regular exercise, avoidance of tobacco and alcohol). However, according to observational research, lower risks of coronary artery disease, obesity, arterial hypertension, type 2 diabetes, and some or perhaps all malignancies are linked with plant-based and vegetarian diets [5,6,7,8,9,10,11,12]. Randomized experiments have indicated that vegetarian and especially vegan diets have a positive influence on a variety of cardiovascular (CV) events [13].

The nutritional sufficiency and quality of plant-based and vegetarian diets should be evaluated individually, not based on how they are labeled, but on the type, amount, and diversity of nutrients ingested [14]. For instance, some studies, although not all [15,16], show that vegans may have poorer bone mineral density and a greater fracture risk due to reduced calcium intake. Individuals who follow a vegan diet may also fail to consume sufficient vitamin B12 and require vitamin B12 supplements [17,18]. The aim of this review is to summarize the contemporary evidence regarding the effect of a vegan diet on human health, underline the links between a vegan diet and cardiovascular diseases (CVD), including both the positive and negative effects of veganism on the CV system, and provide information regarding the potential therapeutic implications of a vegan diet in CVD.

This article summarizes what is known about the impact of a vegan diet on the cardiovascular system. To the best of our knowledge, our article provides a novel insight into this topic by up-to-date information, detailed tables as well as dynamic and more extensive figures. The effect of a vegan diet on the cardiovascular system is also compared to other diets.

## 2. Assessment of Vegan Nutrition concerning Health

Vegetarianism and veganism, which are defined in terms of a low frequency of consumption of animal foods, have grown in popularity and exposure over the last decade due to reasons ranging from environmental to animal welfare concerns, as well as possible health advantages. Vegans, whose numbers are rising in high-income countries across the Western world, make up an increasing fraction of the overall population. Moreover, even though the prevalence of vegans in Europe is estimated to be between 1 and 10%, the precise figure varies in each country, since veganism is primarily associated with religious and ethical views, environmental concerns, and cultural and social values [19].

Many health professional organizations have issued statements on vegan diets. Vegan diets should be properly designed and should be able to offer appropriate nourishment throughout life [20,21]. Even though it was once considered risky to be vegan throughout infancy, childhood, pregnancy, and lactation, it is now established that well-planned vegan diets may provide appropriate and balanced nutrition. Yet, the nutrients consumed on a vegan diet are not always beneficial. Vegans, for example, might eat plant-based meals that are heavy in sugar, salt, or harmful fats [22].

Furthermore, according to current data, the protein ratio of daily calorie consumption is greater in omnivores than in vegans [23]. Nevertheless, a well-planned vegan diet can meet general protein needs. Vegans should boost their protein consumption to 10% of their calorie intake [24]. Amino acids determine protein quality. Plant proteins include all necessary amino acids. While one research study found that combining different protein sources for each meal is not necessary if different plant foods are consumed throughout the day [25], other studies show that ingesting grains (methionine) and legumes (lysine) together delivers better effects on the bioavailability of key amino acids [26,27]. Vegan diets meet protein requirements through nuts, grains, seeds, legumes, green leafy vegetables, pseudo cereals (buckwheat, quinoa), soy, and other derivative products [28].

Vegan diets are beneficial in terms of fiber, beta-carotene, vitamin C and K, folic acid, magnesium, and potassium consumption, making them high-quality diets [29]. Such diets are often rich in ω-6 fatty acids as well [30]. Despite these advantages, they usually tend to feature insufficient intake of vitamin B12, vitamin D, calcium, selenium, zinc, and iodine and fewer accessible ω-3 fatty acids (eicosapentaenoic acid (EPA) and docosahexaenoic acid (DHA)), which can result in energy, nutritional, and micronutrient deficiencies. Therefore, supplementation of these nutrients is essential [31]. The recommended daily intake and supplementation of certain micronutrients in a vegan diet are presented in Table 1.

For instance, in a vegan diet, long-chain ω-3 fatty acids, which are critical for retina, brain, and cell membrane function, can only be consumed as a-linolenic acid (ALA), and it is therefore recommended that vegans take an algae-based DHA dietary supplement in addition to regular dietary intake of ALA sources [32]. Most significantly, approximately 5% to 6% of the daily energy requirement in vegan diets should come from saturated fat, mostly tropical or high-fat foods, according to the American Heart Organization [33].

## 3. Links between a Vegan Diet and Cardiovascular Diseases

### 3.1. Inflammatory Response as a Result of Unhealthy Dietary Patterns

#### Chronic Inflammation of the Vessel Walls Due to Unhealthy Diet

The standard diet that has become widely embraced in many countries over the past 40 years is rather unhealthy, containing relatively high amounts of alcohol [34] and processed foods [35], especially those with additives [36], and only a few fruits and vegetables and other meals rich in fiber and prebiotics [37,38]. An unhealthy diet can alter the structure and function of the gut microbiota and has been linked to increased gut permeability [39,40,41] and epigenetic modifications in the immune system [39], which can lead to low-grade endotoxemia and systemic chronic inflammation (SCI) of the vessel walls [39,40,41]. Similarly, foods with a high glycemic index, such as pure sugars and refined grains, which play an important role in most ultra-processed foods, can lead to increased oxidative stress and thus activate inflammatory genes [42].

Within this framework, uncontrolled alcohol consumption that triggers various reactions in the body causes chronic inflammation to increase over time rather than resolve. In the gut, for example, excess alcohol can lead to the proliferation of bacterial waste products, particularly endotoxins, substances that cause inflammation by activating proteins and immune cells. With more endotoxin production, the inflammation worsens instead of improving [40].

Trans fats, which raise low-density lipoprotein (LDL) and reduce high-density lipoprotein (HDL), and dietary salt (NaCl) are two other components of an unhealthy diet known to be pro-inflammatory. As stated by a recent cohort study of 44,551 French people, a 10 percent increase in the proportion of highly processed food consumption was associated with a 14 percent higher risk of all-cause mortality, consistent with the predicted adverse health outcomes of eating foods high in trans fats and sodium [43].

In this context, according to another observational study of 80,082 women in the Nurses’ Health Study cohort, it was revealed that higher consumption of trans fats, or to a lesser extent saturated fats, is correlated with an increased risk of coronary heart disease (CHD), while higher consumption of polyunsaturated (non-hydrogenated) and monounsaturated fats is associated with a reduced risk [44]. The relationship to CHD risk can only partially be explained by the unfavorable effect of trans fats on the lipid profile.

At the same time, in a cross-sectional study of 730 women from the same patient cohort, markers of endothelial activation such as CRP (C-reactive protein), E-selectin, soluble intercellular adhesion molecule (ICAM-1), and soluble vascular cell adhesion molecule (VCAM-1) were higher, suggesting that increased trans fatty acid intake may promote inflammation and adversely affect endothelial function. CRP levels were 73% higher in women in the highest quintile of trans fat intake than in the lowest quintile [45].

Endothelial dysfunction contributes significantly to the pathogenesis of atherosclerotic cardiovascular disease (ASCVD), which is a chronic inflammatory disease of the arterial wall mediated mostly by phagocytic (eating) leukocytes (white blood cells) such as monocytes and macrophages. This initiates a complicated pathogenic cascade that starts with the accumulation of circulating LDL in the subendothelial layer of the arteries [46,47,48,49]. Following an endothelial injury, LDL becomes mildly oxidized [50]. Further oxidation of LDL leads to fully oxidized LDL (ox-LDL), which is then avidly taken up by macrophages via scavenger receptors (SR) and ultimately transformed into foam cells (the hallmark of early fatty streak lesions) [51].

**Figure 1:**
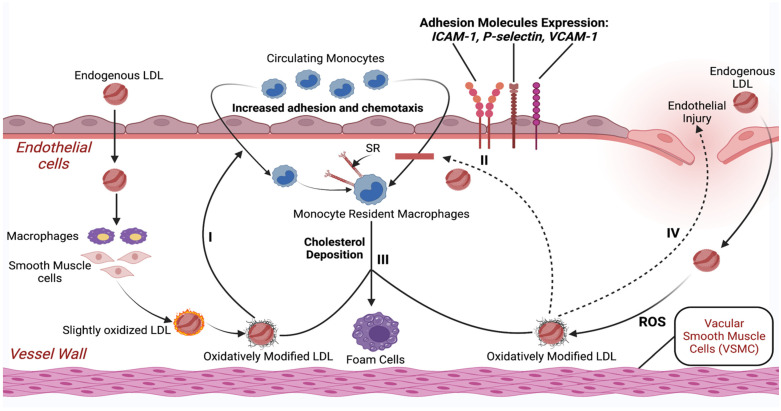
Schematic illustration of endothelial damage and initiation of the atherosclerotic process with only higher LDL levels as a risk factor. Abbreviations: LDL—low-density lipoprotein; ROS—reactive oxygen species; SR—scavenger receptor; ICAM-1—intercellular adhesion molecule-1; VCAM-1—vascular cell adhesion protein-1. Created with BioRender.com (accessed on 17 April 2021).

The expression and detachment of adhesion glycoproteins on the endothelium regulates leukocyte attachment to endothelial cells. The leukocyte binding to endothelial tissue is mediated by the aforementioned types of adhesion molecules (ICAM-1), (VCAM-1), as well as selectins (e.g., P-selectin and E-selectin) [52]. The pathophysiology of endothelial damage and atherosclerosis due to higher LDL levels resulting from an unhealthy diet is illustrated in Figure 1.

Overall, dietary patterns that are high in sugar, alcohol, saturated and trans fatty acids, and refined starches and low in natural antioxidants and fiber from fruits, vegetables, and whole grains may activate the innate immune system, most likely through an increase in pro-inflammatory cytokines and a decrease in anti-inflammatory cytokines. This imbalance may stimulate the formation of a pro-inflammatory state, which causes endothelial dysfunction at the vascular level, resulting in a predisposition to atherosclerotic plaque formation. This can lead susceptible people to an increased prevalence of CHD.

It is vital to understand that both an unhealthy diet and a vegan diet can initiate chronic inflammation if a vegan diet contains inadequate amounts of essential vitamins and nutrients as well as omega-3 fatty acids [31].

### 3.2. Benefits/Risks of a Vegan Diet for the Cardiovascular System

**Figure 2:**
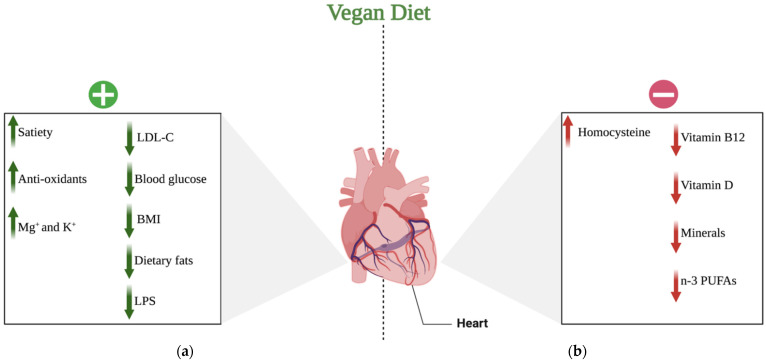
Potential effects of a vegan diet on the cardiovascular system: (**a**) positive effects of a vegan diet on the cardiovascular system; (**b**) negative effects of a vegan diet on the cardiovascular system. Abbreviations: (+) positive; (−) negative; ↑ increase; ↓ decrease; LDL-C—low-density lipoprotein-cholesterol; BMI—body mass index; LPS—lipopolysaccharide; K^+^—potassium cations; Mg^+^—magnesium cations; n-3 PUFAs—n-3 polyunsaturated fatty acids. Created with BioRender.com (accessed on 17 April 2021).

CVD is the leading cause of mortality, currently accounting for one-third of all deaths worldwide and growing in prevalence. Examples of CVDs include CHD, peripheral artery disease, cerebrovascular disease, rheumatic and congenital heart disease, and venous thromboembolism. In this context, vegan diets are considered to improve health and decrease the risk of CVD. On the other hand, according to some studies, a vegan diet may be related to reduced intake of protein, vitamins, or minerals, and thus should also be evaluated in terms of harmful effects. The research on veganism is contradictory and inadequately evaluated [53]. The vast majority of studies on health effects of vegan and plant-based diets were short term and cannot give accurate data on cardiovascular outcomes, which has mostly been estimated based on the changes in biomarker concentrations. More studies with hard endpoints such as major adverse cardiovascular events are required to fully understand the effects of vegan and vegetarian diet on the cardiovascular system. The potential positive and negative impacts of a vegan diet on the CV system discussed in the following paragraphs are shown in Figure 2.

#### 3.2.1. The Positive Effects of Veganism on the Cardiovascular System

PBDs, especially vegan diets, have been shown to provide several health benefits [54]. Numerous studies have demonstrated that the benefits of a vegan diet on human health are due to increased daily consumption of fresh fruits, vegetables, cereal grains, nuts, legumes, and seeds, indicating that vegans make healthier lifestyle choices than those who follow other dietary patterns [55]. Some of the potential health merits include a decreased rate of certain conditions, such as CVD. Vegan diets have been reported to be low-risk therapies for decreasing BMI, systolic and diastolic blood pressure (SBP and DBP), and LDL levels, minimizing the incidence of coronary heart disease events by 40% [56].

Additionally, the results of a systematic review and meta-analysis of cohort studies, which were eventually drawn from the analyses of only two studies, indicated that greater adherence to the PBDs was significantly associated with a lower risk of all-cause mortality (10%) (HR: 0.90, 95% CI: 0.82, 0.99; I^2^ = 90.7%, p_heterogeneity_ < 0.001), and CHD mortality (23%). Based on the same study, it was also found that among vegan diets, compliance with a usual vegetarian diet might protect against CVD and CHD mortality [57].

Because of its low saturated fatty acid (SFAs) and high fiber content, the vegan diet is characterized by low energy intake. Dietary fibers are a diverse group of plant molecules (carbohydrate polymers with ten or more monomer units) with varying physical and chemical characteristics [58]. Water-soluble (SFs) and insoluble fibers (IFs) are the two most common types. Because these fibers are not hydrolyzed by digestive enzymes, they are not completely digested in the human gut [59]. They alter intestinal function by regulating intestinal motions, increasing fecal bulk, and avoiding constipation, among other things. SF, for instance, dissolves in water and forms thick solutions (gels) in the intestinal lumen, delaying or partially reducing carbohydrate, lipid, and cholesterol absorption. These viscous gels can also delay emptying of the stomach and prolong food absorption, enhancing satiety and altering insulin and glycemic post-prandial responses [60].

Moreover, we know that meals of vegetable origin are high in polyphenols, which are natural bioactive chemicals generated by plants as secondary metabolites [61]. Polyphenols may also benefit CV health by inhibiting platelet aggregation, reducing inflammation of the vessel walls, modulating apoptotic processes, lowering LDL oxidation, and improving the lipid profile [62]. Several in vitro investigations have revealed that polyphenols have a high antioxidant capacity due to their ability to neutralize reactive oxygen species (ROS). Their antioxidant properties, presumably paired with their ability to modify nitric oxide (NO) synthesis, allow them to protect endothelial function [63].

Other antioxidant minerals found in a vegan diet include vitamin C, vitamin E, beta-carotene, potassium, and magnesium. Potassium has been proven to decrease blood pressure and the risk of stroke due to its favorable effects on endothelial function and vascular homeostasis [64]. Furthermore, magnesium has been linked to better cardiometabolic outcomes due to its effect on glucose metabolism as well as its anti-inflammatory, vasodilatory, and antiarrhythmic characteristics [65].

The influence on cholesterol metabolism is another important way that a vegan diet might benefit CV health. The low SFA concentration and high unsaturated fat content can enhance the lipid profile. SFAs have been found to activate the pro-inflammatory toll-like receptor-4 (TLR4) signaling pathway, resulting in the production of cytokines capable of triggering a chronic inflammatory state [66,67]. SFAs can also interact with the gut microbiota by facilitating the transfer of lipopolysaccharide (LPS), which is a powerful endotoxin, a mediator of systemic inflammation, and a driver of septic shock [68].

Various studies, however, have revealed that polyunsaturated fatty acids (PUFAs) activate several anti-inflammatory pathways. As a result, a diet high in unsaturated fats and low in SFAs can lower the risk of CVDs through its potential anti-inflammatory effects [69,70].

A meta-analysis of observational studies that compared the vegan diets to omnivorous diets observed that the vegan diet has less energy and saturated fat as a result it protects against cardiometabolic conditions. The authors discovered a decrease in fasting blood glucose and BMI and an improvement in the lipid profile [71].

#### 3.2.2. The Negative Effect of Veganism on the Cardiovascular System

In contrast, lower intake of n-3 long-chain polyunsaturated fatty acids (i.e., DHA and EPA), vitamins (i.e., vitamin B12 and D), specific nutrients, including selenium, zinc, iodine, and calcium, as well as higher levels of essential amino acids (i.e., homocysteine), may explain some of the unfavorable CV effects associated with vegan diets, such as the potential increased risk of ischemic stroke [72]. One study has found that vegans and vegetarians had a higher risk of ischemic stroke than people who ate animal products (HR, 1.54; 95 percent CI, 0.95–2.48) [53].

Nevertheless, systematic reviews and meta-analyses that compared vegetarians and vegans to nonvegetarians has shown no clear association with stroke or subtypes of stroke for vegans and vegetarians [73,74]. At the same time and as specified by a meta-analysis, with a large-scale study design of 657,433 participants, the incidence of total stroke was lower among vegetarians in studies conducted in Asia than nonvegetarians (HR = 0.66; 95% CI = 0.45–0.95; I^2^ = 54%, *n* = 3). Furthermore, the same review found that although there is no strong association between vegetarian diets and a reduced risk of stroke in young adults, there is evidence that older participants aged 50–65 years following a vegetarian pattern exhibited a lower incidence of stroke than nonvegetarians [74].

Although a plant-based diet and especially a vegan diet is believed to be healthy, it can nonetheless result in a higher level of essential amino acids. The most important molecule in this study is homocysteine (Hcy), which has been identified as a risk factor for atherosclerotic vascular disease and hypercoagulability. Given that, there is evidence of a link between hyperhomocysteinemia arising from a vegan diet and CVD, such as heart attacks and strokes [75].

In this regard, and as previously stated, while plant foods provide various nutrients, including dietary fiber and phytochemicals, they do not contain enough vitamin B12 to meet the needs of their consumers, leading to severe deficiency. Deficiency in vitamin B12 will eventually result in hyperhomocysteinemia [76]. Subsequently, because it lowers vascular flexibility and alters homeostasis, elevated homocysteine will induce vascular endothelial impairment. It may also aggravate the negative consequences of risk factors such as hypertension, smoking, cholesterol, and lipoprotein metabolism [77]. Most critically, Hcy has been recognized as a significant CVD risk factor [78].

Besides causing hyperhomocysteinemia, a vitamin B12 deficiency caused by avoiding animal-based meals can also contribute to an increased risk of cardiac conditions via its function in macrocytosis [79]. To be more specific, a deficiency in vitamin B12 can increase the risk of macrocytic anemia, a condition arising from the abnormal growth of red blood cells. In this way, it leads to various diseases, such as heart failure (HF), coronary artery disease, and stroke. It can additionally decrease oxygen delivery and the carrying capacity of blood vessels, undermining circulation’s primary role.

Similarly, vitamin D deficiency, which can be caused by veganism, increases the risk of CVD and is linked to other well-known risk factors for heart diseases such as high blood pressure, obesity, and diabetes [80,81,82]. In particular, low levels of the vitamin may initially predispose the body to congestive heart failure and chronic blood vessel inflammation (associated with hardening of the arteries). It might also change hormone levels, increasing insulin resistance and thus raising the risk of diabetes [83].

To date, certain cross-sectional studies have connected vitamin D deficiency to an increased risk of CVD, such as hypertension, heart failure, and ischemic heart disease [84]. Simultaneously, other prospective studies have found that vitamin D deficiency increases the risk of incident hypertension or sudden cardiac death in individuals who already have CVD [83].

Not only vitamin deficiencies, but other dietary components found in vegan diets can promote an inflammatory response and hence contribute to the development of chronic inflammation [6,85]. These include micronutrient deficiencies, such as zinc and selenium, caused by eating processed or refined foods low in vitamins and minerals, as well as insufficient omega-3 levels, notably EPA and DHA, which impact the resolution phase of inflammation. Eventually, inflammation will induce an atherogenic response, resulting in serious health problems, including arrhythmias, heart failure, and CHD [86].

## 4. Therapeutic Implications

CVD prevention is a major public health concern. It has long been known that vegetarians, and specifically vegans, experience occurrences of chronic CVD, including ischemic heart disease, less frequently [87]. Moreover, it has been demonstrated that incidences of risk factors and comorbidities such as angina and heart failure, hyperlipidemia, diabetes, and obesity, as well as hypertension, can be reduced due to a plant-based diet [56,88]. Even in cases of endothelial damage, a vegan diet is beneficial in slowing or stopping the advancement of coronary atheroma [11,89,90]. These observations have sparked an investigation into the possibility of adopting a vegan diet as a therapeutic tool in preventing or treating CVDs. A summary of clinical studies evaluating relevant outcomes associated with the CV system in patients following a vegan diet is shown in Table 2.

### The Role of a Vegan Diet in the Prevention of CVD

As reported by evidence from interventional trials, a low-fat vegetarian and vegan diet has been effective in treating coronary artery disease (CAD) for over 45 years. Research has suggested that a well-planned plant-based diet is equally as successful at lowering cholesterol as statin medications and a highly advantageous alternative to other treatment strategies [102]. At the same time, it has the distinct benefits of having no side effects or contraindications, is affordable, and has high patient compliance [103].

As aforementioned, vegetarians, particularly vegans, have a decreased incidence of hypercholesterolemia, primarily total and LDL cholesterol [104]. The chief reason for this is that they have increased insulin sensitivity and thus lowered cholesterol production [105]. The fact that vegans have lower cholesterol levels indicates a reduction in the risk of atherogenesis, and consequently CAD. Furthermore, due to their efficiency in eliminating potentially atherogenic remnants, the metabolisms of vegans have been discovered to have superior control of triglyceride-rich lipoproteins [106], thereby exerting protective effects against obesity and the incidence of diabetes.

A study that compared the weight change between patients on a Mediterranean diet and those on a vegan diet discovered that the vegan diet resulted in a 6.0 kg decrease in mean body weight, compared to no change on the Mediterranean diet (treatment effect 6.0 kg (95% CI 7.5 to 4.5); *p* 0.001). Most of the weight loss during the vegan diet was due to the decline in fat mass and visceral fat volume (treatment effect 3.4 kg (95% CI 4.7 to 2.2); *p* 0.001; and 314.5 cm^3^ (95% CI 446.7 to 182.4); *p* 0.001, respectively). In the end, of the 52 study participants, only 26 lost weight on the Mediterranean diet, compared to 48 following the vegan diet [107].

Meanwhile, a recent case report described the effects of a plant-based diet on a 79-year-old patient with diagnosed triple vessel disease (80–95% narrowing) and left ventricular systolic failure (ejection fraction = 35%), all in the setting of worsening dyspnea. Two months on a plant-based diet resulted in clinically significant weight and cholesterol reductions, as well as enhanced exercise tolerance and ejection fraction (+15%) [108]. Other systematic reviews and meta-analyses have shown that vegan diets enhance glycemic management, with substantial reductions in HbA1c levels [109], lowering the chance of developing type 2 diabetes.

Likewise, a systematic review, which assessed how effective the PBDs are for treating obesity and related cardiometabolic health outcomes, stated that plant-based diets demonstrate improved weight control and cardiometabolic outcomes related to lipids, cardiovascular end points, blood pressure, insulin sensitivity, A1C, and fasting glucose, and a lower risk of diabetes compared with usual diets and in some cases standard health-oriented diets such as the American Heart Association (AHA), American Diabetic Association (ADA), and Mediterranean diets. This study suggested that plant-predominant diets practiced as part of sustained lifestyle interventions can stabilize or even reverse DM 2 and CVD [110].

The impact of a vegan diet on blood pressure is important because arterial hypertension is the most common CVD risk factor [111]. The study by Pettersen et al. showed that vegans, lacto-ovo vegetarians, and partial vegetarians had lower estimated odds of arterial hypertension (OR = 0.37; 95% CI: 0.19–0.74; OR = 0.57; 95% CI: 0.36–0.92 and OR = 0.92; 95% CI: 0.50–1.70) than non-vegetarians [112]. In a meta-analysis by Gibbs et al., a vegan diet was not significantly associated with systolic blood pressure (−1.30 mmHg; 95% CI: −3.90,1.29) [113]. In addition, in a meta-analysis of randomized clinical trials by Termannsen et al., no significant effect of a vegan diet on blood pressure was found [114]. In a meta-analysis of randomized clinical trials by Lopez et al., more optimistic results were obtained. A vegan diet was found to significantly reduce systolic (−4.10 mmHg; 95% CI: − 8.14 to − 0.06) and diastolic (−4.01 mmHg; 95% CI: −5.97 to −2.05) blood pressure in subjects with baseline systolic blood pressure ≥ 130 mmHg. In subjects with baseline systolic blood pressure < 130 mmHg, no significant antihypertensive effect of a vegan diet was observed [115]. The results of these studies indicate that the adoption of a vegan diet should be seen as more important in supporting the treatment, rather than the prevention, of arterial hypertension.

Exercise and weight loss are first-line treatments for hypertension at all stages. However, a cross-sectional study indicated that a vegan diet is the most important intervention. This study compared the blood pressure of inactive vegans, endurance athletes consuming a Western diet and running an average of 48 miles per week, and inactive participants that were only consuming a Western diet. Based on the findings of the study, blood pressure was remarkably lower in the vegan group [54].

The positive results associated with the preceding study are most likely attributable to the abundance of antioxidant minerals prevalent in a vegan diet, such as magnesium and potassium. Because of their beneficial effects on endothelial function, and their antiarrhythmic and vasodilatory properties, both potassium and magnesium have been proven to lower blood pressure and thus contribute to the electrical stability of the heart, resulting in better cardiometabolic outcomes [65].

At this point, it is crucial to indicate some of the characteristics and potential differences between pure veganism and other diets with regard to cardiovascular effects. The main differences are shown in Table 3.

## 5. Outlook and Conclusions

PBDs, particularly vegan diets, which exclude all animal-based foods, reflect a feeding pattern that groups have followed for many years, primarily for ethical, ideological, and environmental reasons [19]. Extensive research has been carried out to show the positive as well as negative impacts of a vegan diet on the CV system. Vegan diets are thought to improve health and reduce the risk of CVDs, including CAD, arrhythmias, and heart failure [53]. However, according to some studies, a vegan diet may be associated with lower intake of protein, vitamins, or minerals [116], inducing chronic inflammation and, thus, an atherogenic response.

Finally, a vegan diet is mostly used to improve body weight and composition [117]. Accordingly, this dietary pattern seems to be a feasible and reliable approach to preventing and treating CVD and its risk factors [53] since it minimizes the risk of hypercholesterolemia, hypertension and CAD, type 2 diabetes, and obesity. Especially considering that CAD is such a widespread cause of disability and mortality [118], a well-planned vegan diet combined with appropriate nutritional supplementation might be considered preventative for patients at high CVD risk.

## Figures and Tables

**Table 1:** Summarized daily intake and supplementation of vitamins and minerals in a vegan diet.

Micronutrients	Recommended Daily Intake	Sources in a Vegan Diet	Supplementation
Iron	32 mg (women), 14 mg (men)	Leafy greens (kale, spinach, broccoli, seeds, soybeans)	Yes
Calcium	525 mg	Nuts, kale, seeds, tofu, kidney beans	Yes
Zinc	11 mg (men), 8 mg (women)	Nuts, seeds, whole grain, beans	Yes
Iodine	150 μg	Seaweed, iodized salt	Yes
Vitamin B12	6 μg	Foods enriched with B12 (plant milk, some soy products, and some breakfast cereals)	Yes
Vitamin D	600–800 IU	Fortified soy milk, mushrooms, fortified cereals, fortified orange juice, almond and rice milk	Yes
Omega-3	200–300 mg of DHA	Flax seeds, chia seeds, hemp seeds, walnuts, leafy greens (present as ALA)	Yes

Abbreviations: DHA—docosahexaenoic acid; ALA—alpha-linolenic acid.

**Table 2:** Summary of relevant outcomes and main findings in the included studies.

Authors	Study Information	Intervention Diet	Duration	Outcomes	Main Findings
Kaartinen et al., 2000 [91]	A non-randomized, controlled study including 28 women with fibromyalgia (66% overweight)	Low-salt, lactobacteria-rich vegan diet	3 months	Joint pain, sleep quality, VAS, BMI	↓Weight (BMI = −4 kg/m^2^; *p* = 0.0001)
Barnard et al., 2005 [92]	An RCT including 64 overweight, post-menopausal women	Low-fat vegan diet	14 weeks	Dietary intake, weight, and composition, resting metabolic rate, insulin sensitivity	↓Weight (Weight = −5.8 kg ± 3.2 kg; *p* = 0.012)
Turner-McGrievy et al., 2007 [93]	An RCT including 62 overweight, post-menopausal women	Low-fat vegan diet	14 weeks	Weight loss maintenance and diet adherence	↓Weight (Weight: 1 year = −4.9 kg and 2 year = −3.1 kg; *p* < 0.05)
Elkan et al., 2008 [94]	An RCT including 68 individuals with rheumatoid arthritis	1-day low-energy fast with a gluten-free vegan diet for 1 year	1 year	Blood lipids, oxLDL, anti-PC, BMI	↓Weight (Weight = −4.2 kg; BMI = −1.4 kg/m^2^; *p* < 0.001)
Barnard et al., 2009 [95]	An RCT including 99 T2DM individuals	Low-fat vegan diet with low GI	74 weeks	HbA1c, plasma lipids, weight	↓Weight (Weight: Non-completers = −4.4 kg and completers = −6.8 kg; *p* = 0.25)
Ferdowsian et al., 2010 [96]	A non-randomized, controlled study including 113 overweight and/or pre-existing T2DM patients	Low-fat vegan diet	22 weeks	Body weight changes, anthropometric measures, BP, lipid profile, dietary intake	↓Weight (Weight = −5.1 kg ±0.6 kg; BMI = −2.0 kg/m^2^; *p* < 0.001)
Mishra et al., 2013 [97]	An RCT including 291 overweight and/or T2DM patients	Low-fat vegan diet	18 weeks	Dietary intake, body weight, plasma lipids, BP, HbA1c	↓Weight (Weight: Non-completers = −2.9 kg and completers = −4.3 kg; BMI: Non-completers = −1.04 kg/m^2^ and completers = −1.5 kg/m^2^; *p* < 0.001)
Wright et al., 2017 [98]	An RCT including 65 participants with obesity and T2DM and/or ischemic heart disease and/or hypertension and/or hypercholesterolemia	Low-fat WFPB diet (type of vegan diet)	12 weeks	BMI, cholesterol	↓Weight (Weight: 6 months = −12.1 kg and 12 months = −11.5 kg; BMI: 6 months = −4.4 kg/m^2^ and 12 months = −4.2 kg/m^2^; *p* < 0.0001)
Jakse et al., 2017 [99]	A non-randomized, controlled study involving 325 patients willing to participate after attending a lecture	Low-fat vegan diet followed by supplementation of two daily meal replacements	10 weeks	% Body fat, visceral fat, weight, muscle mass	↓Weight, Body fat + Preservation of muscle mass (Weight = −5.6 kg; *p* < 0.001)
Kahleova et al., 2018 [100]	An RCT including 75 overweight participants	Low-fat, high-carbohydrate vegan diet	16 weeks	Weight, body composition, insulin resistance	↓Weight, ↑Carbohydrate, fiber intake (Weight = −6.5 kg; BMI = −2.0 kg/m^2^; *p* < 0.001)
Barnard et al., 2018 [101]	An RCT consisting of 45 patients with T2DM	Low-fat vegan diet with low GI	20 weeks	Body weight, HbA1c, plasma lipids, urinary albumin, BP	↓Weight (Weight = −6.3 kg; BMI = −2.3 kg/m^2^; *p* = 0.10 for weight and 0.075 for BMI)

Abbreviations: VAS—visual analog scale; BMI—body mass index; RCT—randomized controlled trial; *p*—significance; ↓ decrease; ↑ increase; oxLDL—oxidized low-density lipoprotein; anti-PC—anti-phosphorylcholine; T2DM—type 2 diabetes mellitus; GI—glycemic index; HbA1c—glycated hemoglobin; BP—blood pressure; WFPB—whole-food plant-based diet.

**Table 3:** Comparison between vegan diet and other diets with respect to CV system.

Dietary Pattern	Includes	Outcome and Cardiovascular Effect
Vegan Diet	Vegetables, grains, nuts, fruits, legumes (dried beans, peas and lentils), and foods made from plants	↓SFAs and sodium intake ↓BMI, promotes weight loss ↓blood glucose ↑vasodilation (↓SBP,↓DBP) ↓LDL-C and dietary fats ↓T2DM ↓ApoB and ApoE ↓risk of stroke, LV hypertrophy and LV diastolic dysfunction
Lacto-ovo Vegetarian Diet	Grains, fruits and vegetables, legumes, seeds, nuts, dairy products and eggs	↓SFAs and sodium intake ↓BMI, promotes weight loss ↓SBP ↓LDL-C ↓T2DM ↓risk of heart disease and stroke
Pescatarian Diet	Fruits, vegetables, grains, legumes, eggs, dairy, nuts, seeds, seafood	↓BMI, promotes weight loss ↓BP ↓TC ↓diabetes ↓ heart attacks, atherosclerosis and strokes
Omnivorous Diet	Plants, animals, algae and fungi	↑SFAs and sodium intake ↑BMI ↑SBP ↑LDL-C ↑cancer, diabetes, heart disease
Mediterranean Diet	Whole grains, olive oil, fruits, vegetables, beans and other legumes, nuts, herbs, spices, meat, seafood	↓BMI, promotes weight loss ↓BP ↓LDL-C ↓thrombosis ↓heart disease, stroke
Western (American) Diet	Meat, highly processed foods (high in carbohydrates), dairy products (high fat), grains, refined sugar and saturated fats, low in fruits and vegetables, alcohol	↑SFAs and sodium intake ↑BMI and obesity risk ↑risk of high LDL-C ↑risk of hypertension ↑inflammation, atherosclerosis, oxidative stress ↑heart attack risk (a third of heart attack deaths worldwide)

Abbreviations: ↓ decrease; ↑ increase; SFAs—saturated fatty acids; SBP—systolic blood pressure; DBP—diastolic blood pressure; LDL-C—low-density lipoprotein cholesterol; BMI—body mass index; ApoB—apolipoprotein B; ApoE—apolipoprotein E; LV—left ventricle; T2DM—type 2 diabetes mellitus; BP—blood pressure; TC—total cholesterol.

## Data Availability

Not applicable.
